# Physiological Responses and Salt Tolerance Evaluation of Different Varieties of *Bougainvillea* under Salt Stress

**DOI:** 10.3390/plants13172409

**Published:** 2024-08-28

**Authors:** Di Zhang, Yuan Xue, Ning Feng, Jing Bai, Dexing Ma, Qianqian Sheng, Fuliang Cao, Zunling Zhu

**Affiliations:** 1 College of Landscape Architecture, Nanjing Forestry University, Nanjing 210037, China; zhangdi@njfu.edu.cn (D.Z.); qqs@njfu.edu.cn (Q.S.); zhuzunling@njfu.edu.cn (Z.Z.); 2 Co-Innovation Center of Sustainable Forestry in Southern China, Nanjing Forestry University, Nanjing 210037, China; 3 Jinpu Research Institute, Nanjing Forestry University, Nanjing 210037, China; 4 Digital Innovation Design Research Center, Nanjing Forestry University, Nanjing 210037, China; 5 Jinggu Environmental Construction Co., Ltd., Nanjing 210037, China; 13915981007@163.com; 6 Qingdao Municipal Engineering Design and Research Institute Limited Liability Company, Qingdao 266000, China; qdsz4@126.com (J.B.); madx2004@126.com (D.M.); 7 College of Art & Design, Nanjing Forestry University, Nanjing 210037, China

**Keywords:** *Bougainvillea*, salt stress, antioxidant enzymes, osmotic regulation, comprehensive evaluation

## Abstract

Soil salinization significantly impacts the ecological environment and agricultural production, posing a threat to plant growth. Currently, there are over 400 varieties of *Bougainvillea* with horticultural value internationally. However, research on the differences in salt tolerance among *Bougainvillea* varieties is still insufficient. Therefore, this study aims to investigate the physiological responses and tolerance differences of various *Bougainvillea* varieties under different concentrations of salt stress, reveal the effects of salt stress on their growth and physiology, and study the adaptation mechanisms of these varieties related to salt stress. The experimental materials consisted of five varieties of *Bougainvillea*. Based on the actual salinity concentrations in natural saline-alkali soils, we used a pot-controlled salt method for the experiment, with four treatment concentrations set: 0.0% (*w*/*v*) (CK), 0.2% (*w*/*v*), 0.4% (*w*/*v*), and 0.6% (*w*/*v*). After the *Bougainvillea* plants grew stably, salt stress was applied and the growth, physiology, and salt tolerance of the one-year-old plants were systematically measured and assessed. The key findings were as follows: Salt stress inhibited the growth and biomass of the five varieties of *Bougainvillea*; the ‘Dayezi’ variety showed severe salt damage, while the ‘Shuihong’ variety exhibited minimal response. As the salt concentration and duration of salt stress increase, the trends of the changes in antioxidant enzyme activity and osmotic regulation systems in the leaves of the five Bougainvillea species differ. Membrane permeability and the production of membrane oxidative products showed an upward trend with stress severity. The salt tolerance of the five varieties of *Bougainvillea* was comprehensively evaluated through principal component analysis. It was found that the ‘Shuihong’ variety exhibited the highest salt tolerance, followed by the ‘Lvyehuanghua’, ‘Xiaoyezi’, ‘Tazi’, and ‘Dayezi’ varieties. Therefore, *Bougainvillea* ‘Shuihong’, ‘Lvyehuanghua’, and ‘Xiaoyezi’ are recommended for extensive cultivation in saline-alkali areas. The investigation focuses primarily on how *Bougainvillea* varieties respond to salt stress from the perspectives of growth and physiological levels. Future research could explore the molecular mechanisms behind the responses to and tolerance of different *Bougainvillea* varieties as to salt stress, providing a more comprehensive understanding and basis for practical applications.

## 1. Introduction

Soil salinization is a significant factor that poses harm to the ecological environment, with profound impacts on agricultural production and land resource utilization [1]. Soil salinization poses a global challenge, affecting around 831 million hectares of soil resources across the world. This issue results in annual economic losses in agriculture due to salinity reaching up to $27.3 billion [2,3]. China faces considerable challenges due to its expanding saline-alkali land area, which is mainly concentrated in arid northwest [4]. Due to the extensive burning of fossil fuels and uncontrolled deforestation of forests, global temperatures are rising, and given irrational irrigation systems and water resource management, accelerating evaporation rates. This leads to the accumulation of salt on the soil’s surface, thereby affecting the natural ecology of the soil [5]. As a result of soil degradation, salt stress significantly reduces plant growth and productivity [6]. Restricted growth serves as a direct manifestation of plant response to salt stress [7], as evidenced by decreased plant height, leaf count, stem length, and aboveground dry weight [8,9]. The effect of salt-induced osmotic stress is a reduction in the soil’s water potential, subsequently leading to the diminished water-absorption capacity of plant roots and resulting in physiological drought [10]. Persistent osmotic stress generates ion imbalances within plant tissues, contributing to ion toxicity, nutrient deficiencies, oxidative stress, alterations in cell membrane permeability, metabolic disorders, and accumulation of toxic substances. These factors ultimately impact plant growth, development, and external morphology [11]. Consequently, a comprehensive study investigating plant growth and physiological responses under salt stress, along with an exploration of the mechanisms underlying the effects of salt stress on plant internal metabolism, is of paramount significance in enhancing plant salt tolerance and facilitating the rational utilization of saline-alkali soil.

*Bougainvillea*, a vine-like shrub belonging to the family Nyctaginaceae, comprises 18 species and boasts over 400 ornamental varieties of horticultural value. Originating from South America, it was introduced to Asia and Europe as an ornamental plant at the midpoint of the Middle Ages and is now widely cultivated globally. *Bougainvillea* exhibits a wide range of cultivars and vibrant bracts, rendering it a captivating ornamental plant [12]. However, the plants prefer warm and humid environments and are not cold-resistant. Over the past few decades, research on *Bougainvillea* has delved deeply into aspects such as germplasm resources and propagation techniques. However, there are few reports on the resistance of *Bougainvillea*, and the varieties studied tend to be quite limited [13,14,15]. Therefore, this study selected five *Bougainvillea* varieties as experimental materials in order to observe the changes in growth-based, morphological, and physiological indicators under salt stress. The aim was to analyze the growth and physiological responses, as well as the adaptation mechanisms of Bougainvillea relating to salt stress, and to compare the differences in salt tolerance among the five varieties. This will help identify which varieties are most suitable for application in saline-alkali soils, providing a reference for future horticultural practices and plant selection.

## 2. Materials and Methods

### 2.1. Materials

The five varieties of *Bougainvillea* tested in this study—‘Shuihong’, ‘Lvyehuanghua’, ‘Xiaoyezi’, ‘Dayezi’, and ‘Tazi’—were sourced from Haikou, Hainan. During the summer of 2016, one-year-old stems were selected from three-year-old plants of each respective variety to be used as cuttings. The chosen cutting medium was a 1:1 mixture of peat and perlite. Collected branches were cut into cuttings approximately 7 cm in length, with the top end cut flat about 1 cm above the bud node, and the bottom end cut at a 45° angle. One or two leaves were retained at the top of the cutting, while the rest of the leaves and petioles were completely removed. The cuttings were then quickly dipped in a 1000× diluted solution of Carbendazim for 10 s to disinfect and kill any bacteria. Cuttings were inserted into the medium at a spacing of 4 cm × 7 cm, at an insertion depth of 3 cm, by firmly pressing the medium around the base of the cutting and then thoroughly watering it. During the cutting period, depending on the specific weather and the growth condition of the *Bougainvillea*, cold water was applied moderately every 3 to 7 days to promote rooting and stable growth. In March 2017, the well-grown cuttings with consistent heights and well-developed root systems were transplanted into plastic pots. These pots were 22 cm in diameter at the top, 15 cm in diameter at the bottom, and had a height of 19 cm, as well as drainage holes at the bottom. A substrate mixture of humus soil, garden soil, and sand in a 1:1:1 ratio, with a quantity of 3 kg per pot, was used, and each pot was cultivated with one cutting. The potted seedlings were placed on a seedbed in the greenhouse and subjected to consistent growing conditions with regular maintenance. After four months of cultivation, the salt stress experiment was conducted.

### 2.2. Study Location

The experimental site is located in the greenhouse at the Xashu Experimental Base of Nanjing Forestry University (32°4′43″ N, 118°49′15″ E). This region falls under the North Subtropical Monsoon Climate, which is characterized by pronounced seasonal variations, plentiful rainfall, and ample sunlight. The environmental temperature of the experimental base was maintained at 23–27 °C, with relative air humidity kept between 65% and 75%. The effective photosynthetically active radiation (PAR) was 1000 μmol/m^−2^ s^−1^, accompanied by an average sunshine duration of 2018 h per year and a frost-free period lasting an average of 229 days.

### 2.3. Experiment Design

Seedlings with consistent growth were carefully selected for the water control treatment on July 1, 2017. After one week, on the 7th day, the first salt irrigation was initiated on the seedlings. Subsequently, the second and third salt irrigations were performed on the 10th and 13th day, respectively, to achieve the desired concentration. This experiment involved NaCl stress. It has been determined that *Bougainvillea* cannot grow normally under 0.8% and 1.0% NaCl treatments. Therefore, four concentration gradients were set up [16]: 0.0% (CK), 0.2%, 0.4%, and 0.6%. Each salt concentration treatment had three replicates, with 25 seedlings per replicate. To avoid salt shock, the salt was added in three stages, at concentrations of 1/6, 1/3, and 1/2 of the final concentration. Each pot was irrigated with 200 mL of solution, and a plastic tray was placed at the bottom of the flower pot to collect any runoff, which was then returned to the pot. Prior to the initial irrigation, the substrate was thoroughly rinsed with deionized water to ensure congruency with the experimental design.

The seedling height, ground diameter, and relative leaf conductance of the seedlings were measured and recorded every 9 days. Simultaneously, sampling was conducted by selecting leaves from the upper parts of each plant with consistent orientation. These leaves were wiped dry, placed in self-sealing bags, and stored at −80 °C for further measurement of other physiological and biochemical indicators. Biomass and other parameters were measured during the final sampling. A total of 5 observations were conducted, spanning 45 days.

### 2.4. Experimental Indicators and Measurement Methods

#### 2.4.1. Salinity Damage Level

After salt stress, the damage to the growth indicators, such as new shoots and leaves of the plants, was assessed at 15, 30, and 45 days. According to the severity of salt stress, the damage was divided into groups using the subsequently described eight levels [17] (Table 1).

#### 2.4.2. Physiochemical Factors

Seedling height was measured using a 2 m steel tape measure, and the relative increases in seedling height and stem diameter were calculated. The fresh and dry weights of the roots, stems, and leaves were determined using an electronic balance. Superoxide dismutase (SOD) activity was assessed using the NBT photoreduction method [18], while peroxidase (POD) activity was determined using the guaiacol colorimetric method [19]. Catalase (CAT) activity was measured using the thiobarbituric acid colorimetric method [20]. Leaf cell membrane permeability was evaluated through the immersion method [21]. The content of malondialdehyde (MDA) was determined by the thiobarbituric acid method [22]. Soluble sugar content, soluble protein content, and proline (Pro) content tests were performed following the methods described by Li Hesheng [23].

### 2.5. Data Analysis

Variance analysis, multiple comparisons, correlation analysis, and principal component analysis of various indicators were conducted using Origin 2023 b and SPSS 23.0 statistical software. Statistical data analyses were performed with Excel 2019.

## 3. Results

### 3.1. Effects of Salt Stress on Growth of Bougainvillea

#### 3.1.1. Salinity Damage Symptoms of *Bougainvillea* under Salt Stress

Following exposure to salt stress, significant alterations were observed in the color, morphology, and leaf abscission of the five varieties of *Bougainvillea*. The severity of salinity damage increased across all five varieties as the duration and concentration of salt stress escalated (Table 2). After 15 days of salt stress, ‘Shuihong’, ‘Lvyehuanghua’, and ‘Tazi’ displayed mild salinity damage symptoms, such as partial leaf-yellowing, particularly under high salt concentration (0.6%). However, ‘Dayezi’ exhibited an amplification in the extent of salinity damage as the salt concentration was heightened. Following 30 days of salt stress, all five varieties of *Bougainvillea* exhibited an elevated level of salinity damage. Based on external morphological assessments of salinity damage symptoms, the five varieties ranked in terms of salt tolerance as follows: ‘Shuihong’ > ‘Lvyehuanghua’ > ‘Xiaoyezi’ > ‘Tazi’ > ‘Dayezi’.

#### 3.1.2. Effects of Salt Stress on the Height Growth and Relative Height Growth of *Bougainvillea* Seedlings

**Figure 1:**
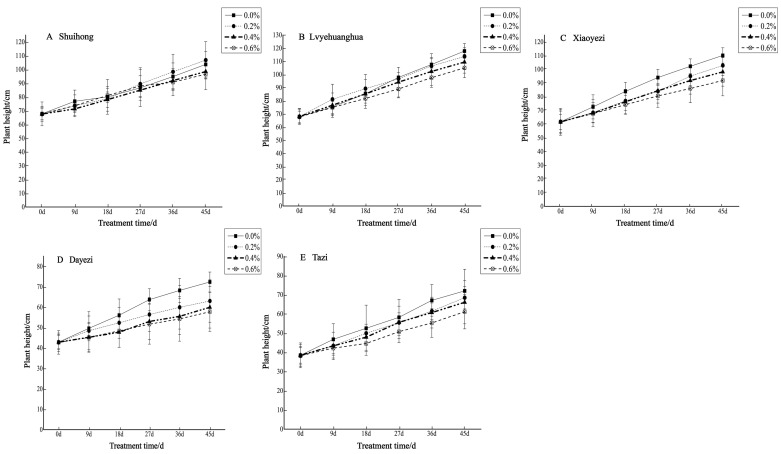
Changes in plant height of five *Bougainvillea* seedlings under different treatment concentrations.

**Figure 2:**
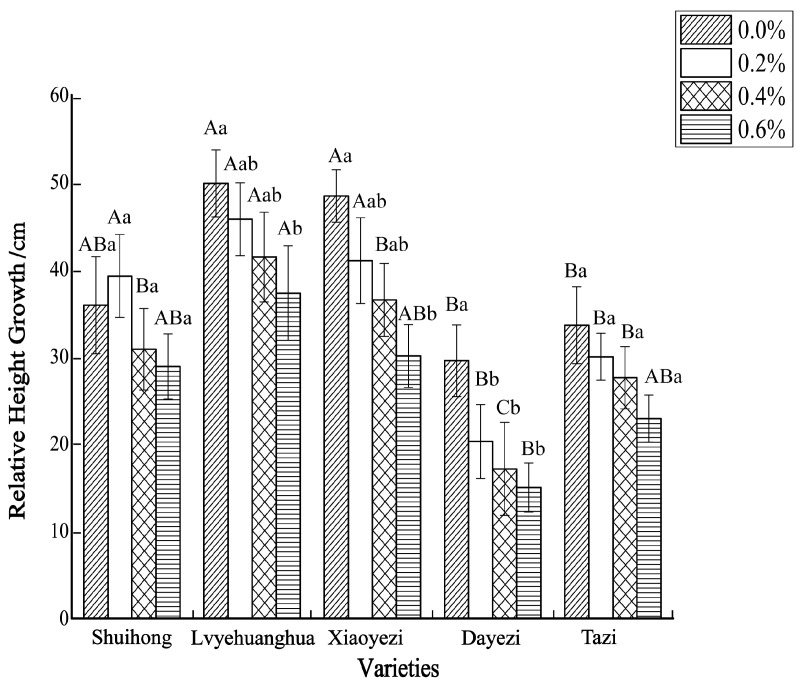
The changes in relative height growth of five kinds of *Bougainvillea* seedlings under salt stress. Note: Lowercase letters in Figure 2 indicate significant differences at the 0.05 level among different concentration treatments on the same variety, while uppercase letters indicate significant differences at the 0.05 level among different varieties under the same concentration treatment.

Salt stress significantly inhibited the growth of the five varieties of *Bougainvillea* (Figure 1), with each variety exhibiting a decreasing trend in relative growth (Figure 2). As the salt concentration increased, the growth rate of each *Bougainvillea* variety gradually declined. After undergoing treatments with different salt concentrations, changes over time did not significantly affect the height growth of the ‘Shuihong’ and ‘Tazi’ seedlings (*p* > 0.05), whereas the height growth of the ‘Lvyehuanghua’ seedlings was significantly affected by the interaction between salt concentration and time (*p* < 0.05). For the ‘Xiaoyezi’ and ‘Dayezi’ varieties, the interaction between salt concentration and time had an even more significant effect on the growth, in height, of the seedlings (*p* < 0.01). The average relative seedling height growth measurements for ‘Shuihong’, ‘Lvyehuanghua’, ‘Xiaoyezi’, ‘Dayezi’, and ‘Tazi’ were 33.71 cm, 43.58 cm, 37.11 cm, 20.48 cm, and 28.52 cm, respectively. It is believed that under salt stress, the growth abilities of the *Bougainvillea* varieties ranked as follows: ‘Lvyehuanghua’ > ‘Xiaoyezi’ > ‘Shuihong’ > ‘Tazi’ > ‘Dayezi’. The two-factor analysis of variance revealed (Table 3) highly significant variations in relative growth among the different varieties (*p* < 0.01), as well as highly significant differences in the effects of various treatments on the relative growth of *Bougainvillea* (*p* < 0.01), while the interaction effect between these two factors was not significant (*p* > 0.05).

#### 3.1.3. Effects of Salt Stress on the Stem Diameter Growth and Relative Ground Diameter of *Bougainvillea*

**Figure 3:**
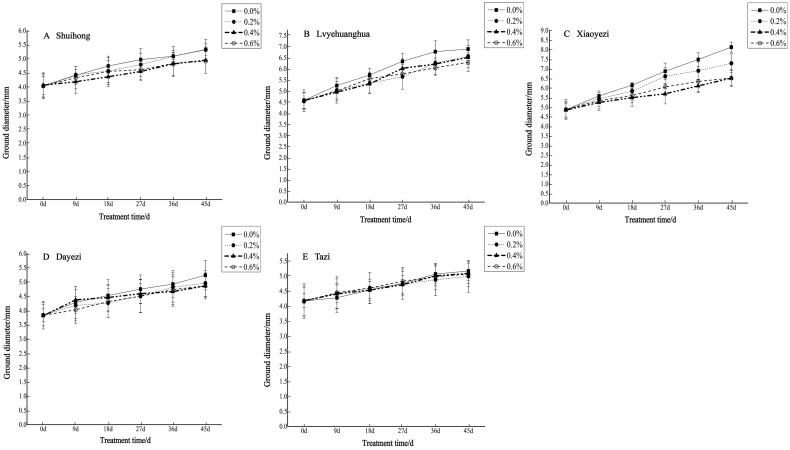
Changes in ground diameter growth of 5 *Bougainvillea* plants under different treatment concentrations.

**Figure 4:**
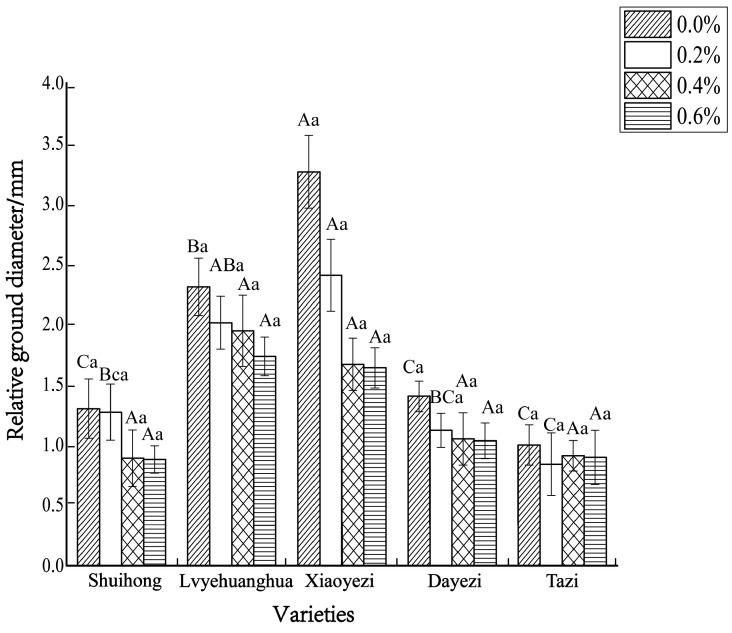
The changes in relative ground diameter of five kinds of *Bougainvillea* seedlings under salt stress. Note: lowercase letters in Figure 4 indicate significant differences at the 0.05 level among different concentration treatments on the same variety, while uppercase letters indicate significant differences at the 0.05 level among different varieties under the same concentration treatment.

The diameter growth pattern of the five varieties of *Bougainvillea* under different salt stress conditions was similar to the seedling height growth pattern (Figure 3). As the duration of salt stress increased, the growth trend of the diameter under high salt concentrations slowed down significantly compared to the control group. The relative ground diameter growth of the five varieties of *Bougainvillea* at different salt concentrations showed minimal changes compared to the control group (Figure 4). Under conditions of different salt concentration treatments, the basal diameter of the five *Bougainvillea* varieties did not exhibit statistically significant interaction effects over time (*p* > 0.05). Significant differences in relative basal diameter among the five *Bougainvillea* varieties were observed under the 0.2% treatment (*p* < 0.05), while differences in relative seedling height among the five *Bougainvillea* varieties were not significant under the 0.4% and 0.6% treatments. The relative ground diameter of ‘Shuihong’ at a 0.2% salt concentration was similar to the control group, at 1.27 mm and 1.30 mm, respectively, and its growth response and relatively high growth rate at low salt concentrations were consistent. The stem diameter growth of ‘Lvyehuanghua’ was at a rate of 9.7% during the early and middle stress periods, but the growth rate decreased in the later period of stress. Among the five varieties, ‘Xiaoyezi’ showed the most significant changes in relative ground diameter; under the treatments of 0.2%, 0.4%, and 0.6%, the relative ground diameter decreased by 26.08%, 48.77%, and 49.69%, respectively, compared to the control. The two-factor analysis of variance (Table 4) revealed a highly significant difference (*p* < 0.01) in the relative ground diameter among different varieties, and the difference in treatments had a significant impact on the relative ground diameter of *Bougainvillea* (*p* < 0.05), but the interaction effect between the two was not significant (*p* > 0.05).

#### 3.1.4. Effects of Salt Stress on the Biomass of *Bougainvillea*

With increasing salt concentration, the root, stem, and leaf dry matter accumulation of the five varieties of *Bougainvillea* exhibited a decreasing trend when compared to the control group (Table 5). The growth of ‘Dayezi’, ‘Tazi’, and ‘Lvyehuanghua’ was considerably more inhibited under high-concentration salt stress. Under the treatments of 0.4% and 0.6% salt concentrations, the total dry weight of ‘Lvyehuanghua’ significantly decreased by 40.33% and 44.26% compared to the control, the total dry weight of ‘Dayezi’ significantly decreased by 33.14% and 44.96% compared to the control, and the total dry weight of ‘Tazi’ significantly decreased by 40.37% and 51.22% compared to the control. Regarding the root-to-crown ratio, values for ‘Shuihong’, ‘Lvyehuanghua’, ‘Dayezi’, and ‘Tazi’ gradually increased with the rising salt concentration. Conversely, the root-to-crown ratio of ‘Xiaoyezi’ demonstrated a declining trend as the salt concentration increased, and all treatment groups exhibited significant differences compared to the control group.

### 3.2. The Impact of Salt Stress on the Physiological Characteristics of Bougainvillea

#### 3.2.1. The Effect of Salt Stress on the Antioxidant Enzyme System in *Bougainvillea* Leaves

**Figure 5:**
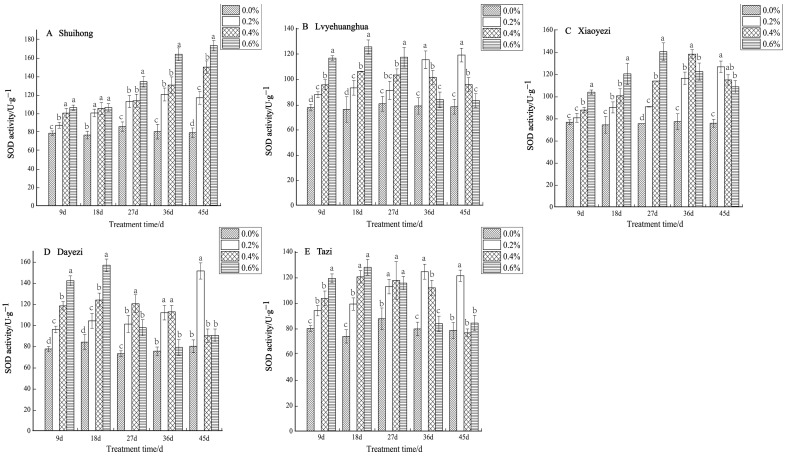
Effects of different concentrations on SOD content in leaves of five kinds of *Bougainvillea.* Note: Different lowercase letters indicate that the differences between treatments at the same times reach the 0.05 significance level.

Under various levels of salt stress, the SOD activity of the five varieties of *Bougainvillea* exhibited, overall, an increasing trend (Figure 5). Specifically, the ‘Shuihong’ variety demonstrated an increase in SOD activity in response to higher salt concentrations. At 9 d and 18 d, there were no significant differences in SOD activity under the 0.4% and 0.6% treatments. However, by 45 days, the differences between treatments were highly significant, with 0.2%, 0.4%, and 0.6% being 147.71%, 189.73%, and 219.22% of the control, respectively. For ‘Lvyehuanghua’, the differences between treatments were relatively significant at 9 d and 18 d, while for ‘Xiaoyezi’, the differences between treatments reached their maximum at 27 d. ‘Lvyehuanghua’ and ‘Xiaoyezi’ displayed a pattern of initially increasing SOD activity followed by a subsequent decrease. Similarly, ‘Dayezi’ and ‘Tazi’ showed comparable changes in SOD activity, with a highly significant increase in SOD activity for both varieties at 9 d and 18 d after salt stress treatment; they reached their peak values at 18 days under the 0.6% treatment, recorded as 156.616 U/g and 127.593 U/g, respectively. As the duration of stress increased, the activity of SOD under each treatment concentration gradually decreased. By 45 d, the highest SOD activity for ‘Dayezi’ and ‘Tazi’ was observed under 0.2% concentration treatment, while the SOD activity under high salt concentrations was reduced and not significantly different from the control. The two-factor analysis of variance (Table 6) outcomes for the differences in SOD among different varieties of *Bougainvillea* are not significant (*p* < 0.01) and the effects of different treatments on the SOD activity of *Bougainvillea* show highly significant differences, while the interaction effect between them is not significant.

**Figure 6:**
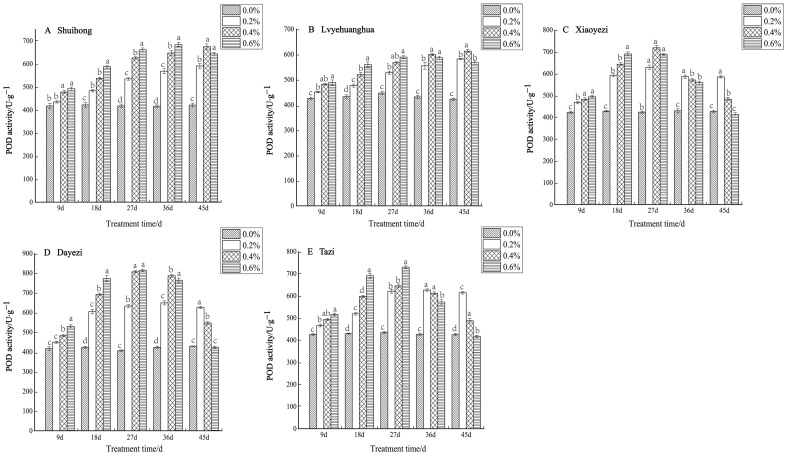
Effects of different concentrations on POD content in leaves of five kinds of *Bougainvillea.* Note: Different lowercase letters indicate that the differences between treatments at the same times reach the 0.05 significance level.

Under various salt concentrations, the POD activity of the five varieties of *Bougainvillea* exhibited an increasing trend (Figure 6). Among the varieties, the changes in POD activity among the treatments at 9 days were smaller compared to the later stages of stress. The POD activity of the ‘Shuihong’ and ‘Lvyehuanghua’ varieties increased with the duration of stress, whereas ‘Xiaoyezi’, ‘Dayezi’, and ‘Tazi’ showed an initial increase followed by a subsequent decline. The ‘Shuihong’ variety reached its maximum value of 683.298 U/g under the 0.6% treatment after 36 days, and the POD activity under different concentration treatments across various stress times showed significant differences compared to the control, while ‘Lvyehuanghua’ displayed a similar pattern of change. ‘Xiaoyezi’, ‘Dayezi’, and ‘Tazi’ exhibited similar patterns as to the changes in POD activity. By day 27 of stress, the POD activity under all treatments increased with the duration of treatment, and then gradually declined. At the end of the stress period (day 45), the activity reached its maximum at the 0.2% treatment level, with values of 584.501 U·g^−^¹, 625.257 U·g^−^¹, and 613.517 U·g^−^¹, respectively. In contrast, the activity of ‘Xiaoyezi’ and ‘Dayezi’ under the 0.6% treatment approached that of the control, showing no significant difference. The activity under high salt concentration (0.6%) was not significantly different from the control. The two-factor analysis of variance (Table 6) indicated no differences in the impacts of different varieties and different treatments on *Bougainvillea*’s POD activity which were highly significant (*p* < 0.01), while the interaction effect between them was not significant.

**Figure 7:**
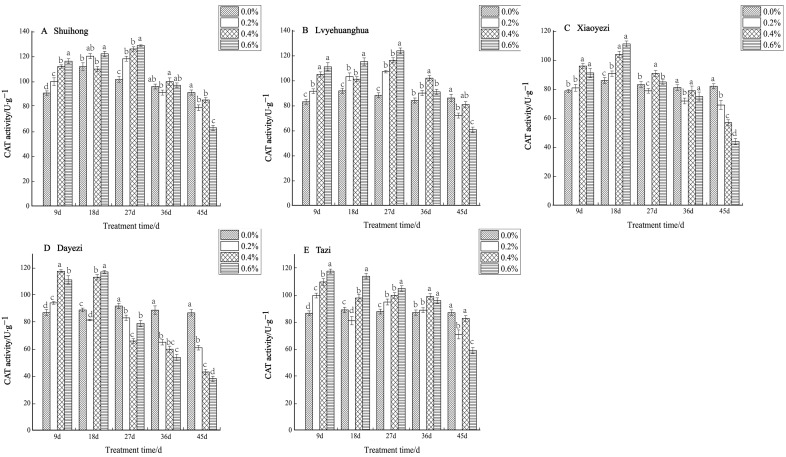
Effects of different concentrations on CAT content in leaves of five kinds of *Bougainvillea.* Note: Different lowercase letters indicate that the differences between treatments at the same times reach the 0.05 significance level.

The CAT activity of five varieties of *Bougainvillea* exhibited an initial increase followed by a subsequent decrease under varying salt concentrations (Figure 7). During the early stage of salt stress, all five varieties demonstrated a significant increase in CAT activity, while in the later stage of stress, the activity gradually declined. The ‘Shuihong’ variety displayed its highest and lowest levels of CAT activity under the 0.6% treatment. At 9 d, 27 d, and 45 d, the CAT activity of each treatment showed significant differences compared to the control. For ‘Lvyehuanghua’, the maximum CAT activity was reached under the 0.6% treatment at 27 d, with noticeable differences between treatments in the early stages of stress. Similarly, ‘Lvyehuanghua’ and ‘Shuihong’ exhibited their maximum and minimum values under the 27-day 0.6% treatment, respectively. The maximum value for ‘Xiaoyezi’ was observed on the 18th day under the 0.6% treatment, at 110.830 U/g, while the minimum value was recorded on the 45th day under the 0.6% treatment, at 43.793 U/g. For the ‘Tazi’ variety, the maximum and minimum values were observed on the 9th and 45th days under the 0.6% treatment. Lastly, the ‘Dayezi’ variety attained its maximum value under the 9-day 0.4% treatment and its minimum value under the 0.6% treatment at the end of the stress period. The two-factor analysis of variance revealed (Table 6) significant differences (*p* < 0.01) in the impacts of different varieties and concentrations on the activity of CAT in *Bougainvillea*, while the interaction effect between them was not significant.

#### 3.2.2. The Effect of Salt Stress on Membrane Permeability and Membrane Oxidative Products in *Bougainvillea*

**Figure 8:**
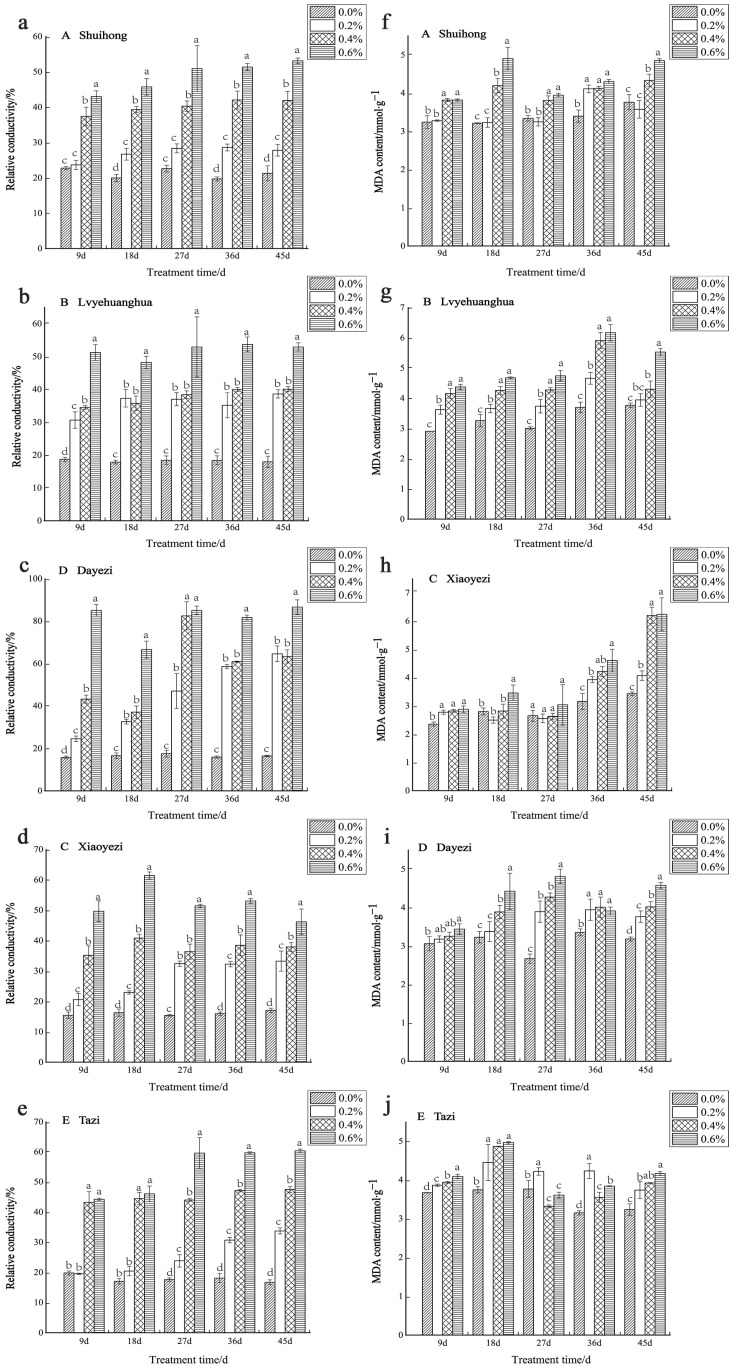
Effects of salt stress on membrane permeability and oxidation products: (**a**) changes in the relative conductivity of ‘Shuihong’; (**b**) changes in the relative conductivity of ‘Lvyehuanghua’; (**c**) changes in the relative conductivity of ‘Xiaoyezi’; (**d**) changes in the relative conductivity of ‘Dayezi’; (**e**) changes in the relative conductivity of ‘Tazi’; (**f**) changes in the MDA content of ‘Shuihong’; (**g**) changes in the MDA content of ‘Lvyehuanghua’; (**h**) changes in the MDA content of ‘Xiaoyezi’; (**i**) changes in the MDA content of ‘Dayezi’; and (**j**) changes in the MDA content of ‘Tazi’. Different lowercase letters indicate that the differences between treatments at the same times reach the 0.05 significance level.

With increases in salt concentration and the duration of stress, the relative conductivity of the five varieties of *Bougainvillea* exhibited an upward trend, revealing significant differences compared to the control group (Figure 8a–e). Among the varieties, in the early stages of stress (9 d), the relative electrical conductivity of ‘Shuihong’ and ‘Tazi’ under low-salt treatment showed no significant difference compared to the control. The ‘Dayezi’ variety demonstrated its highest increase in relative conductivity under high-salt treatment, as recorded at the end of the stress period, reaching 526.24% compared to the control. During the early stages of salt stress, ‘Lvyehuanghua’, ‘Xiaoyezi’, and ‘Dayezi’ exhibited a rapid increase in relative conductivity under the 0.6% treatment. However, as the stress duration was prolonged, the relative conductivity of ‘Xiaoyezi’ decreased. The two-factor analysis of variance (Table 7) indicated that the effects of different varieties and treatments on the relative electrical conductivity of *Bougainvillea*, as well as the interaction effects between them, showed highly significant differences (*p* < 0.01).

With the increase in salt concentration, the MDA content of most *Bougainvillea* varieties generally showed an upward trend. However, some varieties exhibited more complex variation trends at different time points (Figure 8f–j). Specifically, ‘Shuihong’ consistently showed an increased MDA content compared to the control in each treatment period. At 18 days, a significant difference was observed between the high- and low-salt treatments, with its maximum value of 4.91 mmol/g observed under the 0.6% treatment. Meanwhile, ‘Xiaoyezi’ demonstrated an increase in MDA content with the duration of stress, reaching its peak at the end of the stress period. On the other hand, ‘Tazi’ initially experienced a decrease in MDA content at 27 days under high-salt treatment, but later exhibited an increase with increasing treatment concentration at 45 days. The two-factor analysis of variance (Table 7) revealed that the impacts of different varieties and treatments on the MDA content in *Bougainvillea* showed highly significant differences (*p* < 0.01), while the interaction effect between them was not significant.

#### 3.2.3. The Influence of Salt Stress on Osmoregulatory Substances in *Bougainvillea*

**Figure 9:**
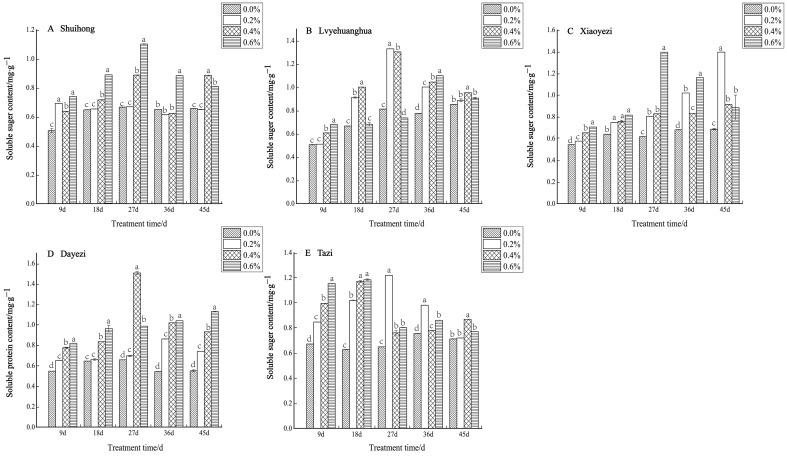
Effects of different concentrations on the soluble sugar content in leaves of five kinds of *Bougainvillea.* Note: Different lowercase letters indicate that the differences between treatments at the same times reach the 0.05 significance level.

With increasing salt concentration and exposure time, the soluble-sugar content of the five varieties of *Bougainvillea* displayed an initial increase followed by a decrease (Figure 9). Specifically, the low-salt treatment (0.2%) significantly augmented the soluble sugar content of ‘Shuihong’ within a short period (9 d), which then stabilized at the level of the control. The highest soluble sugar content was recorded at 1.101 mg/g under the 0.6% salt treatment after 27 days. Likewise, ‘Xiaoyezi’ exhibited an upward trend in soluble sugar content, with increasing stress duration under the 0.2% salt treatment, peaking at 1.395 mg/g after 45 days. ‘Tazi’ also experienced a notable increase in soluble sugar content during the initial stages of stress across various treatments, reaching a maximum of 1.218 mg/g after 27 days. The two-factor analysis of variance analysis outcomes (Table 8) as to the differences in soluble sugar content among different varieties of *Bougainvillea* are not significant (*p* < 0.01), while the effects of different treatments on the soluble sugar content activity of *Bougainvillea* show highly significant differences, though the interaction effect between them is not significant.

**Figure 10:**
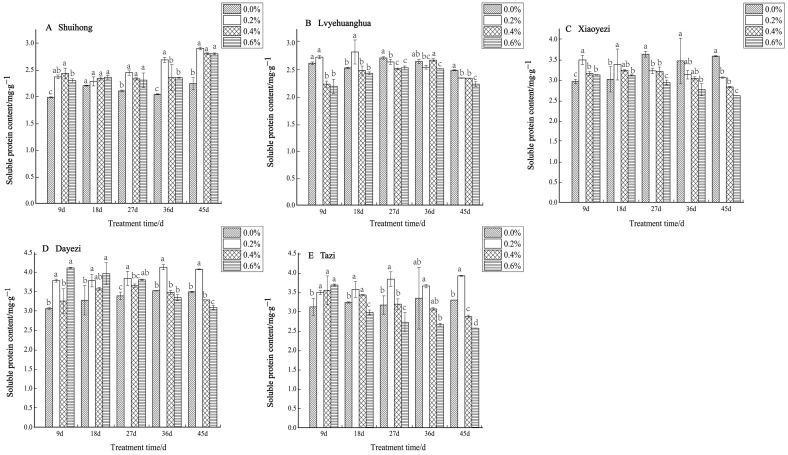
Effects of different concentrations on the soluble protein content in leaves of five kinds of *Bougainvillea.* Note: Different lowercase letters indicate that the differences between treatments at the same times reach the 0.05 significance level.

With the increases in salt concentration and stress duration, the soluble protein content of the five *Bougainvillea* varieties changed, but the response to salt stress varied among different varieties (Figure 10). For ‘Shuihong’, the soluble protein content did not show significant differences at 18 days, while at other time points, the soluble protein content levels under different salt treatments were significantly higher than that of the control. Similarly, ‘Lvyehuanghua’ displayed a gradual increase in soluble protein content during the initial period of salt stress under low salt concentration, reaching a peak of 2.833 mg/g at 18 days under the 0.2% treatment, followed by a gradual decline in soluble protein content observed across all treatments. The soluble protein content in ‘Xiaoyezi’ initially increased and then decreased, at 9 and 18 days, peaking at a 0.2% salt concentration. As the stress duration was extended, the protein content under high salt concentrations (0.4% and 0.6%) decreased. At 9 days, the soluble protein content in ‘Dayezi’ under 0.2% and 0.6% treatments significantly increased, reaching 3.776 mg/g and 4.104 mg/g, respectively. For ‘Tazi’ under salt stress at 9 days, the soluble protein content under each treatment showed a significant increasing trend compared to the control. However, as the stress time continued, the content under the 0.4% and 0.6% treatments gradually decreased, while the content under the 0.2% treatment was highest among its treatments. The two-factor analysis of variance (Table 8) demonstrated highly significant differences (*p* < 0.01) in soluble protein content among different varieties and treatments, as well as a significant interaction effects (*p* < 0.01) between them.

**Figure 11:**
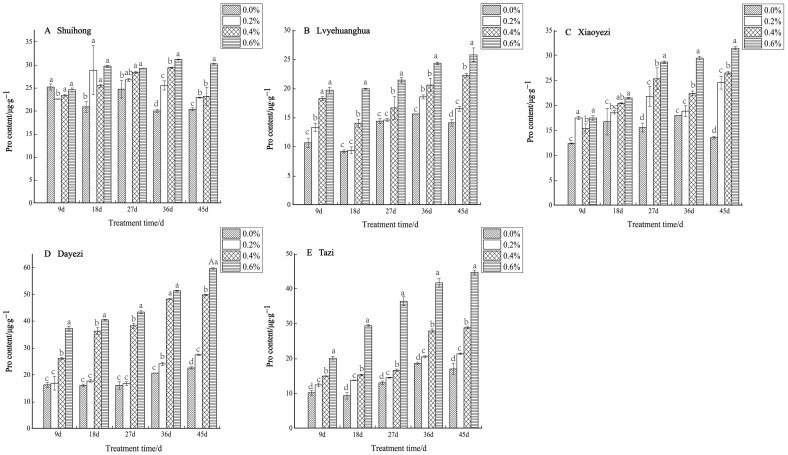
Effects of different concentrations on Pro content in leaves of five kinds of *Bougainvillea.* Note: Different lowercase letters indicate that the differences between treatments at the same times reach the 0.05 significance level.

Under salt stress, the levels of Pro content in the five varieties of *Bougainvillea* showed a consistent increase with both the rise in salt concentration and the duration of stress (Figure 11). Specifically, for the ‘Shuihong’ variety, the Pro content initially decreased and then increased at 9 days, with all concentrations being lower than the control. At 18 days, the Pro content under the 0.2% treatment significantly increased, and all treatments were significantly higher than the control. In the later stages of stress, the Pro content gradually increased with increasing salt concentrations, with the 0.6% treatment showing a relatively high level. At 36 days, the differences between the salt concentrations were quite significant. Conversely, both the ‘Lvyehuanghua’ and ‘Xiaoyezi’ varieties exhibited a significant increase in Pro content as the duration of salt stress increased. Moreover, ‘Dayezi’ demonstrated a notable response to high salt concentrations, with a significant increase in Pro content observed under the 0.4% and 0.6% treatments; at 45 days, the Pro content under 0.2%, 0.4%, and 0.6% treatments was 121.69%, 220.86%, and 264.63% of the control, respectively. For ‘Tazi’, at each treatment time, the Pro content under each treatment showed extremely significant differences compared to the control, with the Pro content under 0.6% treatment being significantly higher than the other treatments and reaching 263.44% of the control at 45 days. The two-factor analysis of variance (Table 8) revealed highly significant differences (*p* < 0.01) in the effects of different varieties and treatments on the Pro content of *Bougainvillea*, as well as significant interactions between these factors (*p* < 0.01).

### 3.3. Comprehensive Analysis of Salt Tolerance in Five Varieties of Bougainvillea

#### 3.3.1. Correlation Analysis of Indicators

**Figure 12:**
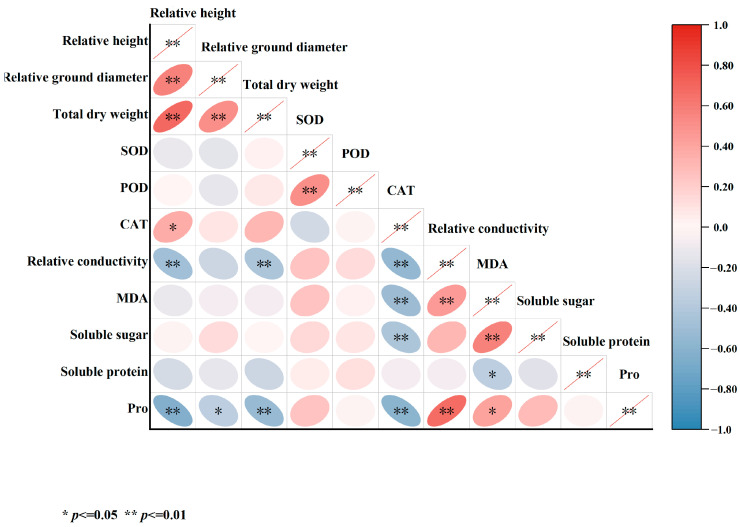
Correlation analysis of various indicators in five varieties of *Bougainvillea* under salt stress.

In this study, a correlation analysis was performed on multiple indicators measured after 45 days of salt stress treatment (Figure 12). The findings indicated significant positive correlations between relative seedling height growth, relative ground diameter, and total dry weight. Notably, total dry weight showed a significant negative correlation with relative conductivity and Pro. CAT exhibited significant negative correlations with relative conductivity, MDA, soluble sugars, and Pro. Moreover, relative conductivity demonstrated a significant positive correlation with MDA and Pro. Additionally, MDA was found to be significantly correlated with soluble sugars.

#### 3.3.2. Principal Component Analysis of Indicators

**Figure 13:**
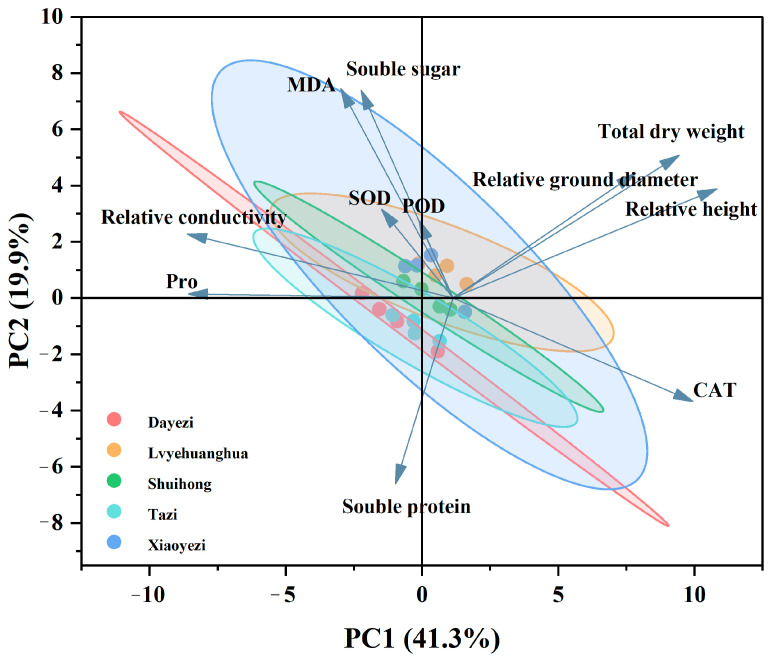
Principal component analysis of various indicators in five varieties of *Bougainvillea* under salt stress.

Principal component analysis was performed on 12 indicators (Figure 13), and the PCA plot shows two main principal components (PC1 and PC2), with a cumulative contribution rate of 61.2%. PC1 accounts for 41.3% of the variance and is primarily influenced by relative plant height, Pro, relative conductivity, total dry weight, and relative stem growth, indicating that PC1 is mainly related to the growth and osmotic regulation system of *Bougainvillea*. PC2, which accounts for 19.9% of the variance, is primarily influenced by MDA, soluble sugars, and soluble proteins, suggesting that the second principal component primarily reflects the physiological stress responses of the plant under salt stress, particularly physiological changes related to membrane permeability and osmotic adjustment.

#### 3.3.3. Comprehensive Evaluation

Based on the results of the principal component analysis of various indicators in the five varieties of *Bougainvillea*, the proportions of the eigenvalues corresponding to the two principal components compared to the sum of the eigenvalues of all extracted principal components were used as weights to establish a comprehensive principal component model. A comprehensive assessment of salt tolerance was conducted; it is presented in Table 9. ‘Shuihong’ achieved the highest comprehensive score (3.370), indicating its high salt tolerance. ‘Lvyehuanghua’ (2.282) and ‘Xiaoyezi’ (1.058) followed, while ‘Tazi’ (−1.478) and ‘Dayezi’ (−2.707) attained the lowest scores, both below 0, signifying their poor salt tolerance. In summary, the ranking of salt tolerance among the five varieties of *Bougainvillea* was as follows: ‘Shuihong’ > ‘Lvyehuanghua’ > ‘Xiaoyezi’ > ‘Tazi’ > ‘Dayezi’.

## 4. Discussion

### 4.1. Response of Bougainvillea to Salt Stress in Terms of Morphology and Growth

The growth morphology of plants is an external manifestation of their physiological metabolic processes. It serves as a visual indicator of their physiological condition and metabolic state [24]. Leaves, as organs responsible for assimilation in plants, are the most immediate and direct components in responding to environmental changes [25]. In this study, with the prolongation of salt-damage time, the leaves of *Bougainvillea* plants exhibited leaf loss, eventually leading to the withering of stems and leaves, and the death of the entire plant. It is speculated that under high salt stress, *Bougainvillea* reduces energy consumption by slowing down or stopping growth to enhance tolerance to stress. At the same time, by accelerating the yellowing and falling of old leaves, the plant reduces the use of energy and transfers the accumulated salts in the aerial parts to the old leaves, thereby reducing the content of Na^+^ and Cl^−^ within the plant. As the degree of salt damage escalates, the morphological appearance of the plants aligns with the research findings of Wang Lei et al. [26].

Similarly, seedling height, stem diameter, and biomass are commonly employed indicators for assessing the extent of salt-induced damage in plants [27]. Seedling height and stem diameter can directly show the growth rate and structural strength of plants, while biomass is a comprehensive indicator for measuring the cumulative growth results of plants. Under salt stress conditions, salinity can interfere with the plant’s water absorption and nutrient balance, leading to impaired physiological and metabolic processes, which in turn affects the growth and development of the plant, and in addition, the slower growth of plants also helps plants survive in unfavorable environments [28]. In this experiment, an increase in both the concentration of salt treatment and the duration of stress resulted in a notable reduction in relative plant height, relative stem diameter growth, and total dry weight across the five varieties of *Bougainvillea*. Nonetheless, variations in the magnitude of decline were observed among the different cultivars, aligning with findings from previous investigations of plants like red maple [29] and sugar beet [30]. This may be due to differences in the response mechanisms associated with salt stress at the physiological and molecular levels among different *Bougainvillea* varieties.

### 4.2. Responses of the Antioxidative Enzyme System and Membrane System of Bougainvillea under Salt Stress

Under salt stress, the antioxidant enzyme systems in plants, including SOD, POD, and CAT, effectively and promptly eliminate reactive oxygen species, minimize damage to macromolecules such as proteins and nucleic acids caused by salt stress, and prevent oxidative harm to membrane systems [31]. Numerous studies have demonstrated that the increased activities of SOD and POD in plants under salt stress are important adaptive mechanisms [32,33]. This increased activity of antioxidant enzymes indicates their capability to counter stress-induced responses. In this study, it was observed that the overall trend in the activity of antioxidant enzymes in plants followed the pattern of an initial rise, followed by a subsequent decline with increasing salt concentration and exposure duration. The speculation is that due to the treatment with low salt concentration, the antioxidant enzyme system in plants actively responds, leading to an elevation in enzyme activity and timely removal of partially generated free radicals [34]. However, as the duration of stress increases, the plant’s capacity for self-regulation diminishes, resulting in a reduction in the activity of the antioxidant enzyme system [35]. Notably, the declining trend in antioxidant enzyme activity becomes more pronounced during the later stages of treatment. In this study, the SOD activity of ‘Shuihong’ showed an increasing trend in the later period, and the change range of POD and CAT was smaller than that of other varieties, so the effect of salt stress on ‘Shuihong’ was less than that of other varieties. Severe stress conditions deactivate the enzyme system in plants, leading to damage of macromolecules such as proteins and subsequently affecting plant growth. The findings of this study align with the results of Sheng Qianqian et al. as to their investigation of NO_2_ stress in *Bougainvillea* [36]. This study did not investigate the non-enzymatic ROS scavenging processes involving antioxidant substances such as AsA and GSH. Future study is suggested in this regard.

The plasma membrane, as the outermost barrier of plant cells, plays a crucial role in plant growth, development, and adaptability to the environment. When plants suffer from salt stress damage, the phospholipid molecules in the plasma membrane are damaged, leading to increased membrane permeability and a large amount of electrolyte leakage. This, in turn, causes changes in electrical conductivity. MDA is one of the products of membrane lipid peroxidation, and its increased content directly reflects the extent of lipid peroxidation. Lipid peroxidation can lead to severe impacts on the structure and function of cell membranes [37,38]. In this study, it was observed that salt stress led to a significant increase in the relative conductivity and MDA content of five varieties of *Bougainvillea* as the salt concentration increased. This indicates that salt stress causes an imbalance in the reactive oxygen metabolism of *Bougainvillea*, significantly increasing the permeability of the cell membranes in the plant leaves, leading to severe damage and destruction. Notably, these results align with a previous investigation on *Pinellia ternate* [39].

### 4.3. Response of Bougainvillea to Osmoregulatory Substances under Salt Stress

In plants, osmolytes involved in osmotic regulation can be categorized as organic and inorganic types [40]. When exposed to adverse stress conditions, plants employ their inherent osmotic regulation mechanisms to adapt to environmental changes and preserve cellular osmotic balance [41]. Under salt stress, soluble proteins and soluble sugars have similar functions. Both can protect the cell membrane by accumulating their own content to coordinate the osmotic pressure balance between the cell and its external environment [42]. This study demonstrated a gradual increase in soluble sugar content and soluble proteins among five varieties of *Bougainvillea* with prolonged salt stress, followed by a slow decline during the later stages of the stress. This observed trend was consistent with changes in soluble sugar content and soluble proteins observed in other plants experiencing adverse stress conditions [43,44,45]. During the initial phases of salt stress, *Bougainvillea* exhibited a notable rise in cellular soluble sugar accumulation, elevating its cytoplasmic concentration to cope with the stress. However, prolonged exposure to stress led to a decrease in soluble sugar content, approaching levels similar to the control group. This suggested heightened cell damage and diminished accumulation of soluble sugars. These findings align with the research conducted by Yan Li et al. on *Michelia maudiae* [46]. Within a certain stress range, plants can resist damage caused by changes in internal osmotic pressure by increasing the content of soluble proteins, thereby enhancing their stress resistance. However, under high levels of salt stress, the organs responsible for protein synthesis in cells may be damaged, leading to a reduction in the synthesis of proteins within the plant [47].

Proline, as an osmolyte, regulates the water potential both inside and outside the cell while safeguarding the structure of macromolecules such as proteins and nucleic acids. This enables plants to cope with salt stress and reduce salt-induced damage [48,49]. This study revealed a notable increase in proline content with elevated salt concentration and prolonged stress duration. The findings demonstrated that as salt stress intensified, proline accumulated substantially, highlighting its robust hydrophilic characteristics which safeguard cell membranes against dehydration damage [50,51], thereby alleviating salt-induced damage in plants.

## 5. Conclusions

The study on the growth and physiological responses of *Bougainvillea* under salt stress led to the following conclusions: (1) growth of all five *Bougainvillea* varieties was inhibited under salt treatment; (2) salt concentration significantly affected the physiological indicators of *Bougainvillea*, with differences among varieties primarily observed in CAT activity, relative electrical conductivity, MDA levels, soluble protein content, and Pro levels; (3) the comprehensive salt tolerance evaluation showed the following order of salt tolerance: ‘Shuihong’ > ‘Lvyehuanghua’ > ‘Xiaoyezi’ > ‘Tazi’ > ‘Dayezi’. These results contribute to the optimization of the cultivation management of Bougainvillea and advance its practical application in saline-alkaline environments.

Due to experimental constraints, this study used only NaCl for single-salt stress treatments, whereas actual saline-alkaline soils typically experience mixed stress and have insufficient soil nutrients. Therefore, future research should conduct field trials in saline-alkaline soils to enhance the comprehensiveness of the experiments. Additionally, future studies could further investigate the molecular mechanisms underlying *Bougainvillea* varieties’ responses to salt stress and their tolerance, providing a more comprehensive understanding and foundation for application.

## Figures and Tables

**Table 1:** Salinity damage levels and symptoms.

Salinity Damage Levels	Characteristics
0	No obvious symptoms of salinity damage
1	A very small number of leaf tips and edges turn yellow
2	Some leaf tips and edges turn yellow, and a few leaves have yellow spots
3	A small portion of the leaf tips and edges turn black and wither
4	Some leaf tips and edges curl and wither, and the leaves fall off
5	Most of the leaves have burnt and curled leaf tips and edges, and some leaves have fallen off
6	Some branches wither, leaves fall off, and a few die
7	Most branches wither, leaves fall off, and some die

**Table 2:** Salinity damage levels exhibited by five varieties of *Bougainvillea* after salt stress.

Types	NaCl Concentration	Investigation Time and Plant Stress Symptoms		
		15 d	30 d	45 d
Shuihong	0.0%	0	0	0
	0.2%	0	0	1
	0.4%	0	1	2
	0.6%	1	2	4
Lvyehuanghua	0.0%	0	0	0
	0.2%	0	0	1
	0.4%	1	3	4
	0.6%	1	4	5
Xiaoyezi	0.0%	0	0	0
	0.2%	0	1	2
	0.4%	1	2	4
	0.6%	1	3	5
Dayezi	0.0%	0	0	0
	0.2%	1	2	5
	0.4%	2	5	6
	0.6%	3	6	7
Tazi	0.0%	0	0	0
	0.2%	0	1	2
	0.4%	0	2	5
	0.6%	1	3	6

**Table 3:** Two-way ANOVA of *Bougainvillea* varieties and salt concentration on the relative seedling height of *Bougainvillea*.

Measurement Indicators	F Value		
	Varieties	Salt Concentration	Interaction Effect
Relative height growth	11.970 **	5.542 **	0.205

Note: the significance of each factor is indicated by *. ** means *p* < 0.01.

**Table 4:** Two-way ANOVA of *Bougainvillea* varieties and salt concentration in relation to the relative stem growth of *Bougainvillea*.

Measurement Indicators	F Value and Its Significance		
	Varieties	Salt Concentration	Interaction Effect
Relative stem growth	14.347 **	3.954 *	0.837

Note: the significance of each factor is indicated by *. ** means *p* < 0.01, * means *p* < 0.05.

**Table 5:** Effects of salt stress on dry weight (g) and root–shoot ratio of different parts of 5 *Bougainvillea* plants.

Types	NaCl Concentration	Root (g)	Stem (g)	Leaf (g)	Total Dry Weight (g)	Root-to-Shoot Ratio
Shuihong	0.0%	0.566 ± 0.023 a	18.060 ± 0.866 a	15.824 ± 0.460 b	34.450 ± 1.188 b	0.017 ± 0.001 b
	0.2%	0.571 ± 0.008 b	18.501 ± 0.371 b	12.891 ± 0.694 b	31.962 ± 0.883 c	0.018 ± 0.000 a
	0.4%	0.509 ± 0.006 c	12.906 ± 0.358 c	12.784 ± 0.760 c	26.200 ± 1.010 d	0.020 ± 0.001 a
	0.6%	0.437 ± 0.030 a	10.869 ± 0.260 a	11.071 ± 0.013 a	22.376 ± 0.217 a	0.020 ± 0.002 c
Lvyehuanghua	0.0%	0.729 ± 0.028 a	20.761 ± 0.167 b	19.428 ± 0.310 a	40.918 ± 0.384 a	0.018 ± 0.001 c
	0.2%	0.755 ± 0.038 a	21.587 ± 0.059 a	17.446 ± 0.325 b	39.788 ± 0.338 a	0.019 ± 0.001 bc
	0.4%	0.565 ± 0.067 b	13.248 ± 0.128 c	10.603 ± 0.392 c	24.416 ± 0.271 b	0.024 ± 0.003 ab
	0.6%	0.554 ± 0.040 b	12.433 ± 0.289 d	9.822 ± 0.364 d	22.808 ± 0.449 c	0.025 ± 0.002 a
Xiaoyezi	0.0%	1.539 ± 0.029 a	15.742 ± 0.495 a	10.657 ± 0.412 b	27.938 ± 0.856 b	0.058 ± 0.002 a
	0.2%	1.217 ± 0.038 b	16.384 ± 0.131 a	11.550 ± 0.108 a	29.150 ± 0.264 a	0.044 ± 0.001 b
	0.4%	0.944 ± 0.022 c	13.532 ± 0.408 b	9.661 ± 0.096 b	24.137 ± 0.482 c	0.041 ± 0.002 b
	0.6%	0.807 ± 0.014 d	13.480 ± 0.033 b	8.404 ± 0.310 c	22.691 ± 0.329 d	0.037 ± 0.001 c
Dayezi	0.0%	0.732 ± 0.020 a	9.601 ± 0.290 a	7.152 ± 0.162 a	17.485 ± 0.354 a	0.044 ± 0.001 a
	0.2%	0.740 ± 0.051 a	9.525 ± 0.071 a	7.020 ± 0.426 a	17.285 ± 0.390 a	0.045 ± 0.004 a
	0.4%	0.524 ± 0.008 b	6.726 ± 0.207 b	4.442 ± 0.309 b	11.691 ± 0.299 b	0.047 ± 0.002 a
	0.6%	0.437 ± 0.031 c	5.420 ± 0.094 c	3.407 ± 0.192 c	9.624 ± 0.254 c	0.050 ± 0.005 a
Tazi	0.0%	0.732 ± 0.022 a	9.619 ± 0.094 a	8.343 ± 0.325 a	18.693 ± 0.364 a	0.041 ± 0.002 c
	0.2%	0.539 ± 0.017 bc	5.168 ± 0.107 b	5.669 ± 0.325 b	11.376 ± 0.235 b	0.050 ± 0.001 b
	0.4%	0.556 ± 0.030 b	5.335 ± 0.161 b	5.256 ± 0.438 bc	11.146 ± 0.284 b	0.052 ± 0.003 b
	0.6%	0.514 ± 0.010 c	3.890 ± 0.076 c	4.716 ± 0.140 c	9.119 ± 0.073 c	0.060 ± 0.001 a

Note: Different lowercase letters indicate that the differences between treatments on the same variety are significant at the 0.05 level.

**Table 6:** Two-way ANOVA of *Bougainvillea* varieties and salt concentration on the antioxidant enzyme system of *Bougainvillea*.

Measurement Indicators	F Value and Its Significance		
	Varieties	Salt Concentration	Interaction Effect
SOD	2.017	23.326 **	0.739
POD	1.812	23.480 **	0.501
CAT	10.016 **	23.772 **	1.333

Note: the significance of each factor is indicated by *. ** means *p* < 0.01.

**Table 7:** Two-way ANOVA of *Bougainvillea* varieties and salt concentration as to the membrane permeability and oxidation products of *Bougainvillea*.

Measurement Indicators	F Value and Its Significance		
	Varieties	Salt Concentration	Interaction Effect
Relative conductivity	20.699 **	155.190 **	4.642 **
MDA	3.867 **	14.151 **	0.627

Note: the significance of each factor is indicated by *. ** means *p* < 0.01.

**Table 8:** Two-way ANOVA of *Bougainvillea* varieties and salt concentration as to the osmoregulatory substances of *Bougainvillea*.

Measurement Indicators	F Value and Its Significance		
	Varieties	Salt Concentration	Interaction Effect
Soluble sugar content	2.072	11.690 **	1.351
Soluble protein content	98.189 **	11.053 **	3.072 **
Pro	22.679 **	42.808 **	4.768 **

Note: the significance of each factor is indicated by *. ** means *p* < 0.01.

**Table 9:** Ranking of comprehensive salt tolerance scores among the five varieties of *Bougainvillea*.

Varieties	Scores	Ranking
Shuihong	3.370	1
Lvyehuanghua	2.282	2
Xiaoyezi	1.058	3
Tazi	−1.478	4
Dayezi	−2.707	5

## Data Availability

The datasets generated during (and/or analyzed during) the current study are available from the corresponding author on reasonable request.
